# Emerging roles of chromatin in the maintenance of genome organization and function in plants

**DOI:** 10.1186/s13059-017-1236-9

**Published:** 2017-05-23

**Authors:** Zaida Vergara, Crisanto Gutierrez

**Affiliations:** Centro de Biología Molecular Severo Ochoa, CSIC-UAM, Nicolas Cabrera 1, Cantoblanco, 28049 Madrid, Spain

## Abstract

Chromatin is not a uniform macromolecular entity; it contains different domains characterized by complex signatures of DNA and histone modifications. Such domains are organized both at a linear scale along the genome and spatially within the nucleus. We discuss recent discoveries regarding mechanisms that establish boundaries between chromatin states and nuclear territories. Chromatin organization is crucial for genome replication, transcriptional silencing, and DNA repair and recombination. The replication machinery is relevant for the maintenance of chromatin states, influencing DNA replication origin specification and accessibility. Current studies reinforce the idea of intimate crosstalk between chromatin features and processes involving DNA transactions.

## Introduction

The nuclear processes that are involved in DNA transactions include complex mechanisms responsible for DNA replication, repair, and recombination (the so-called 3Rs). However, the substrate for these processes is not the naked DNA molecule, but chromatin, a highly structured and dynamic macromolecular entity formed by the association of genomic DNA with histones and non-histone proteins. As a consequence, intimate connections exist between these three basic processes and chromatin structure and dynamics. The chromatin status is equally relevant for transcription, another DNA-based process. This process is highly related to the linear topography of different chromatin states and to the three-dimensional (3D) organization of the genome, which defines territories such as euchromatic and heterochromatic domains.

The nucleosome, which is the structural unit of chromatin, consists of a core of eight histone molecules (two each of H2A, H2B, H3, and H4) and 147 bp of DNA wrapped around it. In addition, histone H1 binds to the linker DNA between nucleosomes and plays a crucial role in chromatin compaction [1]. The exchange of canonical histones with variant forms, for example, replacing canonical H3.1 with variant H3.3, contributes to a very significant increase in the diversity of nucleosome types present in the genome [2–4]. Another element of profound structural and functional relevance is the variety of post-translational modifications that occur in residues located in the histone tails [5, 6]. These modifications include acetylations, methylations, phosphorylations, ubiquitylations, sumoylations, carbonylations, and glycosylations [5]. In addition to histone modifications, the DNA can be methylated at C residues, with relevant effects on gene expression [7].

In recent years, advances have been made in our understanding of the complex crosstalk between chromatin, transcriptional activity, genome replication, and repair, as well as in characterizing heterochromatin boundaries. Here, we discuss this progress, with an emphasis on plants, and refer to the interested reader to comprehensive reviews for further details.

## Genome topography

The original observation of distinct sub-nuclear territories, such as the densely condensed regions in the nucleus (chromocenters) [8], has advanced in recent years with the generation of genome-wide maps of dozens of DNA and histone modifications. Multiple combinations of chromatin marks actually occur, so the combinatorial possibilities at a given genome locus are extraordinary. The use of sophisticated computational approaches has not only confirmed the preferential association of certain chromatin marks on a genome-wide scale, but also made it possible to begin to decode the different patterns of DNA and histone modifications across the genome. This work has now been completed in recent years for various eukaryotic model genomes, including those of mammal models [9–12], *Drosophila melanogaster* [13, 14], *Caenorhabditis elegans* [15], *Arabidopsis thaliana* [16, 17], and *Zea mays* [18].

### Linear topography

In Arabidopsis, initial studies that focused on chromosome 4 clearly distinguished four major chromatin states, each with a characteristic combination of histone modifications [16]. Importantly, these chromatin domains, which were scattered along the genome, represented active and repressed genes in euchromatin, silent heterochromatin, and intergenic regions. A more recent study, using genome-wide epigenetic datasets, data on DNA properties such as the GC content, and information on the relative enrichment in canonical histone H3.1 and variant H3.3, identified nine distinct chromatin states defining the entire Arabidopsis genome [17]. These states include those previously reported [16] plus others covering those typical of proximal promoters, transcription start sites (TSS), distal intergenic regulatory regions, and two types of heterochromatin.

**Fig. 1:**
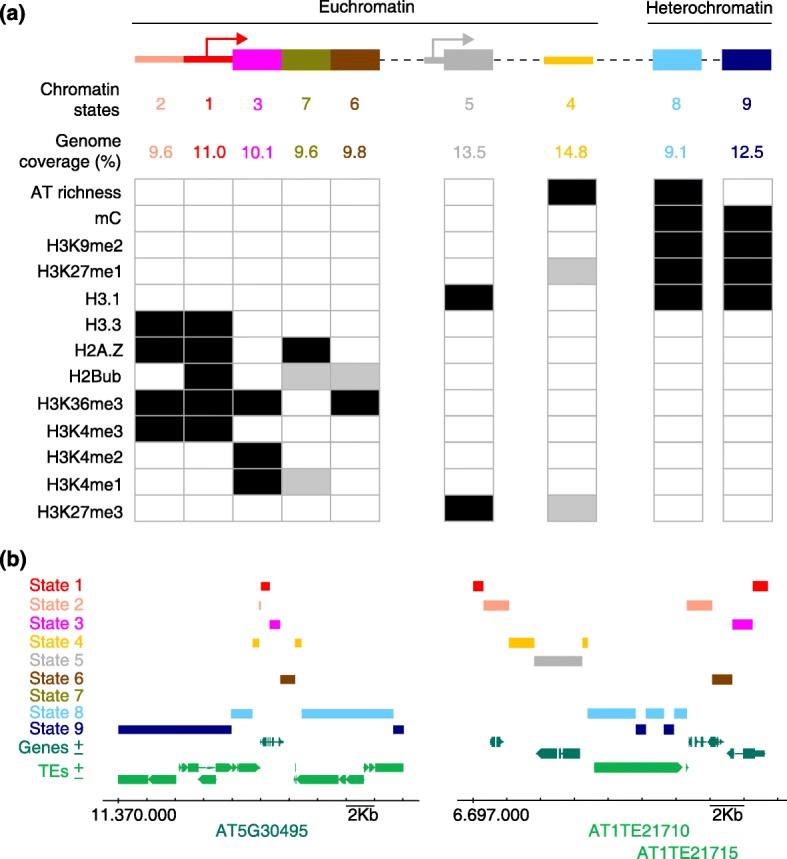
**a** The major genomic elements have distinct chromatin states, each characterized by a signature of chromatin marks: proximal promoters (state 2), TSS (state 1), 5′ end of genes (state 3), long coding sequences (state 7), 3′ end of genes (state 6), polycomb chromatin (state 5), distal regulatory intergenic regions (state 4), AT-rich heterochromatin (state 8), and GC-rich heterochromatin (state 9). The occurrence levels of the main chromatin marks that define each state are summarized as follows: high (*black*), medium (*grey*), very low or absent (*empty box*). **b** Transitions from euchromatin to heterochromatin states. *Left*: example of a highly expressed *Arabidopsis* gene (AT5G30495) that is flanked by repressed transposable elements (*TE*s) in a pericentromeric region of chromosome 5. *Right*: example of contiguous TEs (AT1TE21710 and AT1TE21715) flanked by expressed genes in one arm of chromosome 1. Note that, in both cases, the transition from repressed heterochromatin (states 8 and 9) to the active euchromatin (state 1) occurs through a defined path of other chromatin states

The number of possible chromatin states depends on how many variables are considered in the analysis, and it is expected to increase in the future. However, it is remarkable that the current set of chromatin states represents the five major elements that form the genome (Fig. 1a):

1. Proximal promoters and TSS/5′ UTRs (chromatin states 2 and 1, respectively) are typically characterized by marks that are associated with open and highly accessible chromatin, such as H3K4me2/3, high histone H3.3 and H2A.Z, and low H3.1, and that include highly accessible DNase I sites [19]. A high abundance of H3K36me3 and H2Bub serves to distinguish chromatin state 1 from state 2.
2. The genic regions, including the 5′ end, the 3′ end and the long coding sequences, are defined by chromatin states 3, 6, and 7, respectively. The 5′ end of genes is characterized by relatively high levels of H3K4me1/2 and very low amounts of H3K27me3, whereas at the 3′ end the H3K4me2 modification is almost absent. The coding sequences of long genes may have limited amounts of H3K4me1.
3. The distal regulatory intergenic regions (chromatin state 4) are relatively small due to the compact nature of the Arabidopsis genome, as is also the case in D. melanogaster and C. elegans. Intergenic domains contain moderate levels of H3K27me1 and H3K27me3 and tend to be AT-rich. This domain is also likely to contain many binding sites for transcription factors that act at a distance from the TSS, as recently reported for EIN3 in ethylene signaling [20]. These regions, together with those of chromatin state 2, frequently have properties of bivalent chromatin, containing both H3K4me3 and H3K27me3. Interestingly, these bivalent chromatin regions have been identified in the cells of Arabidopsis plants but only in embryonic animal cells [21]. Full understanding of the biological relevance of this combination of marks awaits a gene-by-gene detailed analysis.
4. Polycomb chromatin has a quite distinct state (state 5), which covers around 13.5% of the Arabidopsis genome, roughly coinciding with the transcription unit, and is highly enriched in H3K27me3 and histone H3.1.
5. Silent heterochromatin, which is enriched in H3K9me2, H3K27me1, and histone H3.1, among other marks, can be separated into AT-rich (chromatin state 8) and GC-rich (chromatin state 9) heterochromatin. Both forms are largely confined to pericentromeric regions, although there are also scattered patches of heterochromatin in the euchromatic chromosome arms.

A detailed analysis of the neighborhoods in which the nine chromatin states occur revealed the occurrence of prevalent associations. As a consequence, a consensus motif could be deduced that defines the linear topography of the major elements in the Arabidopsis genome (Fig. 1a): promoter and TSS (states 2–1), transcription units (states 3–7–6), Polycomb (state 5), distal regulatory intergenic regions (state 4), and heterochromatin (states 8–9). Remarkably, these associations between domain and chromatin state also correlate nicely with the genomic function of each domain.

### Boundaries between chromatin states

As briefly mentioned above, the chromatin states that define the Arabidopsis genome are non-randomly arranged. It is striking that the propensity of a given state to locate in contact with another is highly dependent on its chromatin signature. Thus, TSS (chromatin state 1) is in contact exclusively with states 2 and 3 (proximal promoters and the 5′ end of genes, respectively). This might be expected, but in other cases, the relationships between chromatin states is surprising. For instance, Polycomb chromatin (state 5) is almost exclusively associated with distal regulatory intergenic regions (state 4), which also contain moderate levels of H3K27me3, and with the relatively AT-rich heterochromatin (state 8), but not with GC-rich heterochromatin (state 9). Analysis of the linear relationship among all of the chromatin states clearly revealed that chromatin state 4 behaves as a general hub that serves to connect the other chromatin states (equivalent to genomic elements) and that separates the three major chromatin domains: genic regions, Polycomb chromatin, and heterochromatin. In other words, the transition of one of these domains to another does not occur abruptly but rather through a defined and progressive change in chromatin signatures [17]. Interestingly, this also seems to occur in other genomes, such as that of D. melanogaster [14], but the panorama of chromatin states within genomes that share a less compact organization is not currently known.

Arabidopsis has a small and relatively compact genome in which about 36% of genes are close or immediately adjacent to transposable elements (TEs) [22, 23]. TEs are genomic elements that must be maintained in a silenced and heterochromatic state in most plant tissues, developmental stages, and growth conditions [24, 25]. The constitutive heterochromatic regions are located at the pericentromeric sites, at telomeres, and in the nucleolus organizing regions [26–28]. In addition, there are non-expressed domains within the euchromatic arms that are defined as heterochromatin (that is, enriched in repressive marks). These regions are composed mainly of TEs, inserted within euchromatic regions, and of the polycomb-related genes [26, 29].

The physical barriers between heterochromatin and euchromatin form chromatin boundaries, and in Arabidopsis these often occur in the pericentromeric regions. The presence of these boundaries is considered to be a major component of the linear topography of eukaryotic genomes. There are cases in which (i) highly expressed genes are embedded in the highly repressed pericentromeric heterochromatin and flanked by TEs (Fig. 1b, left panel) or (ii) TEs, with the typical repressed chromatin state, are scattered along the euchromatic chromosome arms (Fig. 1b, right panel). As mentioned earlier, the transition from silent heterochromatin to active euchromatin (e.g., from state 9 to state 1) does not occur abruptly, but through other chromatin states that cover a relatively small boundary region [17]. Whether a single chromatin mark or a combination of marks defines certain genomic locations as boundaries between euchromatin and heterochromatin is not presently known.

From a mechanistic point of view, different processes have evolved to avoid the spreading of heterochromatin into euchromatin. TE silencing in Arabidopsis results from a combination of the activities of C methylation pathways that depend on MET1 [30], CMT2/3 [31, 32], and DRM2 as part of the RNA-dependent DNA methylation (RdDM) pathway [33]. (See Box 1 for expansion of abbreviated gene names used in this review.) In addition, the association of heterochromatin domains with the LINC (linker of nucleoskeleton and cytoskeleton) complex in the nuclear periphery is a spatial component that is relevant for heterochromatin silencing, as revealed using loss-of-function mutants [34]. The RdDM pathway, which relies on RNA Pol IV-dependent 24-nucleotide short interfering RNAs (siRNAs) [35–37] and RNA Pol V-dependent RNAs [38], is crucial for both preserving the boundaries of heterochromatin domains and keeping TEs silent across generations [18, 39–41]. It has recently been found that the RNA polymerase Pol V is directly involved in defining the edges of TEs. Thus, Pol V transcribes short TEs across their entire length, whereas longer TEs produce Pol V transcripts only at their edges [40]. RNA Pol IV transcripts are also associated with TEs but include both the edges and the TE bodies. More importantly, Pol V, but not Pol IV, transcripts show a high strand preference, being generated from the sense strand at the 5′ end of TEs and from the antisense strand at their 3′ ends [40]. These data strongly support the idea that Pol V plays a direct role in defining the heterochromatin boundaries.

In animals, certain histone modifications and related proteins are also involved in defining heterochromatin boundaries; for example, H3K9me2/3 and HP1 occur at the sites of constitutive heterochromatin and H3K27me3 and the PRC2 complex at facultative heterochromatin [42]. In fission yeast, the HP1 homolog (Swi6) is responsible for preventing the heterochromatic boundaries of the pericentromeric regions, but not of the telomeres, from spreading to the neighboring euchromatic genes [43]. There is evidence that this mechanism also operates in plants. For example, the demethylase IBM1 protects against spreading heterochromatin; in the absence of IBM1, active genes are methylated in the CHG context and accumulate H3K9me2 in gene bodies [44] due to the action of KYP and CMT3 [45]. Mutations in the H3K9 methylases, as well as in the LDL2 demethylase, increase H3K4me1 levels in TEs, a prerequisite for TE derepression [44]. Thus, the balance between H3K9me2 and H3K4me1 appears to be crucial in mediating heterochromatin silencing.

Chromosome 4 of *A. thaliana* (Col-0 ecotype) contains a heterochromatic knob in its short arm, although other accessions, such as Ler, are knobless. The knob was generated by a paracentric inversion, involving two VANDAL5 TEs and two F-box genes, that generated new boundaries between heterochromatin and euchromatin. Studies of DNA methylation, histone methylation, and gene expression have revealed that the epigenetic marks are not modified at the newly generated borders. Instead, the inversion causes linkage disequilibrium with the *FRIGIDA* gene in the 132 knob-containing accessions identified [46]. Depending on the distance from the insertion of a TE to a gene, the TE can cause heterochromatic signatures to spread to euchromatic genes. This process has been called position-effect variegation in Drosophila [47]. In *A. thaliana*, this process is known to occur in some genes within the heterochromatic knob of chromosome 4. Some of the genes within the knob remain euchromatic and active, whereas others that are close to a VANDAL TE are silent in wild-type plants and active in the *ddm1* mutant background [48]. Rice artificial tetraploids show a significant increase in DNA methylation of the CHG and CHH contexts that is associated with DNA TEs. More importantly, these DNA methylation changes, linked to alterations in the siRNAs of the RdDM pathway, lead to the repression of genes close to the TEs [49]. The downregulation of these genes, directed by neighbor TE hypermethylation, suggests a possible mechanism for the handling of gene-dosage effects in polyploid plants.

In plant species whose genomes are larger and more complex than that of *A. thaliana*, the association of TEs with euchromatic domains is more frequent. This is the case, for example, in maize, which has a high TE content and in which >85% of genes have a TE within a distance <1 kb [50]. In both maize and Arabidopsis, genes are frequently flanked by a relative increase in mCHH, the least common mC form in genomes, which are known as mCHH islands [51–53]. Recent studies have revealed that these mCHH islands play a crucial role in defining the gene/TE boundaries in >50% of maize genes [18]. Interestingly, mCHH islands are mostly located near the inverted repeats of TEs, in particular at the TE edge close to the gene. As this association is more frequent in expressed genes, there is a possibility that different mechanisms for defining gene–TE boundaries may operate depending on the transcriptional status of the affected gene, but it is also clear that the TEs themselves may affect the transcriptional activity of the gene. Studies in maize have demonstrated the role of mCHH in tagging TE edges near active genes [18]. Thus, mutants that have defects in the *MOP1* and *MOP3* genes, which encode homologs of the Arabidopsis RDR2 and the large subunit of Pol IV, respectively, are deficient in RdDM and in setting appropriate boundaries that prevent an active chromatin state from invading a nearby TE, and vice versa. Furthermore, some maize retrotransposon families show a greater propensity to spread than others, in particular when they are close to genes that are expressed at low levels, pointing to an additional regulatory layer in the control of gene expression [54].

### Nuclear territories

The advances in sophisticated microscopy procedures and analysis, together with recently developed genomic approaches, are contributing to expanding our view of nuclear organization beyond the linear topography of the genome. The so-called 3C (chromosome conformation capture) strategy [55] allows the identification of interactions between one genomic site and many others, and several other genomic procedures have also been developed. These include the 4C (circular chromosome conformation capture) strategy [56], which determines the interaction of one viewpoint with many genomic locations; the 5C (3C carbon copy) strategy [57], which allows the use of many viewpoints; and the Hi-C strategy [58], which is designed to determine the genomic interactions of all loci. The reader is referred to comprehensive reviews for extended discussion of these procedures [59–63]. Here, we highlight only the major discoveries derived from high-throughput genome analysis of chromatin interactions in Arabidopsis [64–68].

**Fig. 2:**
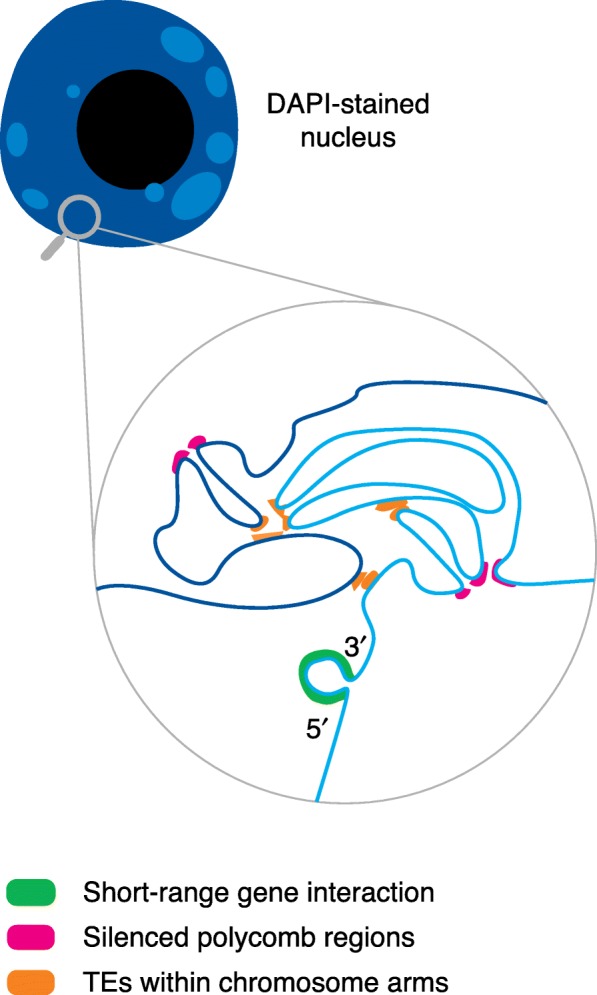
Summary of various types of interactions that determine the spatial organization of nuclear territories, as revealed by Hi-C strategies

A first conclusion of these studies is that the overall 3D interaction network within the Arabidopsis nucleus resembles that of Drosophila and mammalian cells [69] and reveals distinct types of interactions between chromatin states [70]. This is particularly striking for the separation between euchromatin and heterochromatin [64]. In addition, Hi-C experiments identified genomic regions that have the general features of active chromatin that establish distal interactions with other similar domains. Short-range interactions also occur between the 5′ and the 3′ end of genes, in particular in highly expressed genes [67]. One largely studied example of such interactions occurs at the *FLC* locus [71]. By contrast, genomic domains that have the global properties of repressed chromatin establish contacts with similar regions and are separated from active domains [65, 66]. Remarkably, heterochromatic regions—enriched in TEs, H3K9me2 [65], and H3K27me1 [64]—that are interspersed along euchromatic chromosome arms tend to contact each other both in *cis* and in *trans*. This leads to the formation of a specific heterochromatin region, called a KNOT [64]. Interestingly, other repressed regions that establish long-range interactions have been reported to contain promoters that are enriched in the H3K27me3 Polycomb mark [67]. This suggests that such interactions might contribute to the coordinated expression of those genes. A recent study, based on the known interaction of the LHP1 protein with H3K27me3 chromatin, has demonstrated that most of the long-range interactions lost in the *lhp1* mutants showed reduced H3K27me3 levels. This, together with expression analysis, revealed the importance of the interaction of LHP1 with H3K27me3 marks in the 3D organization of the Arabidopsis genome and in the coordination of gene expression [68]. The map of spatial interactions in the Arabidopsis genome is increasingly complex, and it has been proposed that plant chromatin adopts various conformations that involve both short-range and long-range interactions (Fig. 2). The various types of looping, including 5′–3′ loops and enhancer–promoter loops, as well as the factors affecting chromatin architecture over short- and long-ranges have been reviewed in detail recently [72].

The formation of genome territories that are well separated by TADs (topologically associating domains), as described for Drosophila (~100 kb) and mammalian cells (1 Mb) [73–75], does not seem to be a characteristic of the Arabidopsis genome. Owing to the similar sizes of the Arabidopsis and Drosophila genomes, it is perhaps unlikely that the size and compactness of the Arabidopsis genome is the reason for the apparent lack of TADs. Instead, the lack of TADs might be a consequence of the lack in plants of a structural homolog of CTCF in mammals and CP190 in Drosophila [73–75], the proteins that serve as an insulator that defines TAD boundaries [76, 77]. Although typical TADs are missing from Arabidopsis, regions with functional similarities have recently been reported in this plant [63, 66]. Therefore, it could be very interesting to determine how these TAD-like regions are established and whether they are developmentally regulated or respond to hormonal and environmental cues.

## DNA transactions

Basic cellular processes that are involved in the maintenance and transmission of genetic information actually deal with chromatin, not just naked DNA. Thus, the DNA replication, transcription, repair, and recombination machineries have to act on genome regions containing nucleosomes and a plethora of different histone modifications. They need a strict crosstalk with the specific complexes responsible for the disassembly of nucleosomes and their assembly once the process is completed [78]. In addition, the chromatin landscape affects the activity of these macromolecular complexes, which, in turn, also interact with chromatin-modifying complexes. Here, we briefly discuss recent advances on this topic, emphasizing their relevance for genomic and epigenetic maintenance.

## Genome replication and chromatin silencing

The maintenance of epigenetic states is a key aspect of the genome replication process; for example, establishing transcriptional silencing once the replication fork has passed certain genomic regions [79–81]. This silencing is required because histones that are newly deposited by the replicative histone chaperones (CAF-1, NAP1, NRP1) do not contain the same set of post-translational modifications present in parental histones. In some cases, they are actually different isoforms, such as canonical H3.1 (as opposed to variant H3.3) because this is the only H3 deposited by CAF-1 during replication and repair. Remarkably, several components involved in the elongation step during DNA synthesis are directly implicated in transferring epigenetic information to the newly synthesized daughter chromatin strands.

The DNA polymerase α, in complex with DNA primase, is responsible for the synthesis of Okazaki fragments in the lagging strand [82], as well as of the first initiation event in the leading strand in each replication origin (ORI). Its large subunit, POLA1, is encoded by the Arabidopsis *ICU2* gene [83] and forms a complex, most likely at the replication fork, with CLF and EMF2, components of the PRC2 complex that trimethylates H3 at residue K27 [83]. As a consequence, hypomorphic mutations of the *ICU2* gene exhibit altered H3K27me3 levels in numerous PRC2 target genes, including the most studied *FLC*, *FT*, and *AG* [84]. POLA1 acts in concert with ROS1, a methylcytosine DNA glycosylase [85, 86], to regulate silencing of other loci [87].

DNA polymerase δ is the holoenzyme complex that extends the lagging strand [82]. POLD1, the large catalytic subunit of this polymerase, is required to maintain correct H3K4me3 levels of certain flowering genes, including *FT*, *SEP3* [88], and probably many others, by mechanisms that are still poorly known. The second largest subunit, POLD2, is also important for the maintenance of transcriptional silencing [89], suggesting that it is the holoenzyme that participates in maintaining a correct balance of H3K4me3 and H3K27me3. This silencing pathway is independent of changes in methylcytosine levels but, interestingly, is dependent on ATR. In fact, *pold2-1* mutants are defective in the DNA damage response (DDR) after methyl methanesulfonate (MMS) treatment [89].

DNA polymerase ε is the third polymerase at the replication fork responsible for the elongation of the leading strand [82]. Its catalytic subunit, POLE1, which is encoded by the *POLE1*/*ABO4*/*TIL1*/*ESD7* gene in Arabidopsis [90–92], interacts with CLF, EMF2, LHP1, and MSI [93]. As a consequence, POLE1 participates at the replication fork in the maintenance of the H3K27me3 silencing mark in target genes, including flowering genes such as *FT* and *SOC1*, in much the same way as other DNA polymerases. Altered function of DNA Pol ε in hypomorphic mutants of the large subunit or as achieved by altering the levels of the accessory subunit DPB2 results in hypersensitivity to aphidicolin and hydroxyurea. DPB2 overexpression triggers the expression of DNA repair hallmark genes and produces S-phase lengthening, probably leading to partial genome replication [94]. Genetic analysis has revealed that the DNA Pol ε-dependent pathway is coordinated with ATR, SOG1, and WEE1 to respond to replicative stress [95]. Together, all the data available for various DNA polymerases indicate that the molecular complex responsible for the maintenance of epigenetic states and genome integrity is the whole replisome.

Silencing of TEs that are associated with genome replication occurs through a different molecular pathway. It requires the ATXR5/6 histone methyltransferases that generate H3K27me1 specifically in heterochromatin [96, 97]. They exhibit a specific activity on the canonical histone H3.1, which is enriched in TEs [98, 99], owing to steric constraints [100]. The *atxr5*;*atxr6* double mutants have defects in controlling DNA replication, as revealed by their abnormal DNA content profiles, which are indicative of DNA over-replication in peri- and nonpericentromeric heterochromatin [101]. This defect occurs preferentially in tissues containing endoreplicating cells, such as cotyledons and old leaves [101, 102]. The double effect of *atxr5*;*atxr6* mutants in transcriptional silencing and DNA replication is an example of replication–transcription coupling. However, a puzzling observation is that the replication phenotype is suppressed by mutations in the methylcytosine machinery [103], whereas the TE reactivation phenotype is enhanced by the same mutations [102]. This suggests that the transcriptional defects may not be the cause of the replication defects. In fact, decreasing levels of H3K27me1 lead to massive TE transcriptional reactivation resulting from the derepression of TREX activity, which causes an unscheduled excess of transcription to enter into conflict with the replication machinery [102]. One possibility is that an increase in R-loop formation, which has otherwise been linked to the initiation of DNA replication [104], produces replication stress and genome instability.

Biochemical experiments using a whole set of purified yeast replication factors, histones, and chromatin remodeling complexes have directly shown that chromatin organization in the parental strands has profound effects on genome replication efficiency. This occurs at different levels, including ORI selection, the early initiation steps and the replication fork rate [105, 106]. These experiments demonstrate that the presence of nucleosomes in the parental strands determines various parameters that are crucial for DNA replication. Nevertheless, the existence of different types of nucleosomes, depending on their content in canonical and variant histone forms and on the presence of multiple histone modifications, probably has distinct consequences for the replication process. As discussed earlier, these variables lead to a large combinatorial complexity that has been simplified using computational approaches to identify different chromatin states that are characterized by specific signatures in plants [17] and animals [12, 14, 15]. This information will be instrumental in defining the chromatin landscape of individual ORIs showing different states across the genome. An answer to the question of whether ORIs are associated with one or more chromatin signatures awaits the identification of the entire ORI set (the “originome”) in a whole organism.

### Genome repair and recombination

The DDR includes, as a first step, the recognition of the DNA lesion. Accessibility to the damaged site is of primary importance and it is significantly affected by the local chromatin landscape. The DDR triggers a cascade of events that lead to the activation of genes required for various forms of DNA repair, depending on the type of DNA damage and the cell cycle stage, among other factors. Both aspects (accessibility and signaling) have been discussed in a comprehensive manner recently [26, 107–109]. Here, we focus on the newest results, with emphasis on how repair and recombination relate to chromatin and vice versa.

The changes in the H3 and H4 acetylation patterns that occur soon after X-ray irradiation are a direct indication of DDR at the level of histone modifications, as demonstrated by mass spectrometry [110]. The intimate crosstalk between DDR factors and epigenetic information is relevant during initial DDR events. It was unexpectedly found that plants carrying defects in chromatin remodeling complexes or DNA methylation, such as *ddm1* or *ros1* mutants, are also defective in the repair of UV-B DNA damage [111]. Likewise, new roles have recently been found for DDB2, a primary component of the pathway repairing UV-induced DNA damage at the genome level [112]. DDB2 depletion leads to methylation alterations predominantly as the result of a deregulation of the de novo cytosine methylation at centromeric and pericentromeric regions [113]. This is the result of the combined action of (i) DDB2 binding to AGO4, which controls the formation of the 24-nucleotide siRNAs through the RdDM pathway, and (ii) regulation of the expression of the DNA methylcytosine glycosylase ROS1 by DDB2 [113]. Conversely, mutations in *DDM1* lead to hypersensitivity to certain DNA-damaging agents [114].

The upregulation of DNA-repair genes is one of the first readouts of DDR activation. ChIP assays have revealed that the increase in gene expression occurs concomitantly with the increase in H3K4me3 levels, particularly around the TSS and gene bodies, without changes in the DNA methylation levels [115]. The gene expression changes in response to DNA damage are not affected, even after knocking out the six genes encoding NAP1 and NRP histone chaperones [116]. This indicates that they participate downstream in the pathway, probably during nucleosome remodeling associated with DNA repair. It has been shown that NAP1 and NRP are required to trigger homologous recombination (HR) before chromatin is remodeled at damaged sites, once γ-H2A.X foci are formed and in an INO80-dependent manner [116]. Recent results show that NRP1 accumulates in chromatin after DNA damage and binds cytochrome c [117] through the NRP1 histone-binding domain [118]. This interaction is important for NRP1 recycling during the disassembly and reassembly of nucleosomes during DNA repair, which parallels the situation with SET/TAF-1β [119, 120], the animal functional homolog of Arabidopsis NRP1.

These results are in line with others demonstrating that chromatin remodeling complexes, such as SWR1, which is responsible for depositing H2A.Z, also are relevant for efficient DNA repair, as demonstrated by the reduced levels of repair by HR and the hypersensitivity to DNA-damaging treatments of mutants in which its subunits are defective [121]. It must be emphasized that HR is a very risky process when it occurs in heterochromatin because of the high content of repeated sequences. However, HR predominates over non-homologous end joining (NHEJ) in heterochromatin [26]. One possible way to reduce potential conflicts is to translocate the damaged sites outside the heterochromatin domains, as reported in yeast [122]. However, recent data reveal that Arabidopsis has evolved an alternative pathway whereby pericentromeric heterochromatin undergoes significant remodeling as a consequence of DNA damage produced by over-replication, as, for example, in the *atxr5*;*atxr6* mutant. This allows the formation of unique “over-replication-associated centers”, which have an ordered structure consisting of condensed heterochromatin in the outer layer, the H2A.X variant in another layer, and a core containing γ-H2A.X and RAD51, possibly among other DNA-repair factors [123]. A recent report strongly suggests evolutionary differences between plants and animals in the H2A proteins associated with DNA repair. Repair of double-strand DNA breaks (DSBs) in the heterochromatin of mammalian cells depends on the phosphorylation of HP1 and KAP1 [124], whereas a different mechanism operates in plants. Thus, in plants, euchromatin DSB repair depends on H2A.X phosphorylation, whereas in heterochromatin repair this role is played by a specific H2A.W7 protein, which is located exclusively in heterochromatin [125] and is phosphorylated by ATM [126].

A correct epigenetic landscape is also necessary for the highly specific recombination events that take place during meiosis. Thus, the level of cytosine methylation strongly affects recombination at crossover hotspots in different ways: (i) RdDM represses crossover formation in euchromatin, increasing nucleosome density and H3K9me2, and (ii) MET1 represses crossover formation in euchromatin and facilitates crossover formation in heterochromatin, as revealed using *met1* mutant plants [127].

HR is also a survival mechanism that responds to altered DNA replication fork progression. It requires the correct function of DNA polymerase complexes, as revealed recently for POLD2 and the flap endonuclease FEN1 [89, 128]. The preferential nucleolar accumulation of FEN1–GFP poses the question of whether this endonuclease plays a role in genome stability that is related to the organization and copy number of rDNA repeats, an aspect that has not been addressed fully.

## Outlook

Genome organization and function depend heavily on local chromatin properties. The linear topography of chromatin states reveals highly preferred neighborhood associations for the different chromatin states. Why is this necessary and how these preferences are maintained are unanswered questions. In addition, the linear topography facilitates a higher level of complexity by establishing specific domains that have been shown to interact preferentially and to generate a specific organization of nuclear territories in space. Does this simply reflect a structural element of genome organization? Or does it have functional consequences? At least in the case of plants, which have high growth plasticity, it is conceivable that the organization of nuclear domains may change in response to hormonal signals, developmental cues, or environmental challenges. Thus, it is known that the nuclear architecture is modified in response to light during postembryonic development, when heterochromatin reorganization and transcriptional reprogramming are associated with the establishment of photosynthesis [129]. Likewise, epigenetic silencing of TEs is released upon various types of stress, suggesting that the specific chromatin landscapes of silenced TEs, and possibly genes, may regulate their transcriptional response to stress [130].

There are different developmental transitions that are associated with changes in chromatin marks, such as the establishment of a seedling after seed imbibition, the vegetative to reproductive transition, or gametophyte formation. All of these examples rely on changes in H3K27me3 that depend on PRC2 complexes [131]. The gametophytic stage is particularly attractive because of its haploid nature as it is not known whether chromatin states and the organization of nuclear territories depend on ploidy level. Plants contain several dozens of cell types that make up all of their different organs. The individual transcriptomes of all of these cell types have not been obtained yet, but a fair amount of data are becoming available [132–134]. As the transcriptome and the epigenome are intimately linked, the question is whether chromatin states have certain cell-type-specificity. Likewise, a pertinent question is whether changes in the linear topography of the genome have any consequences in the 3D organization of the nucleus. This is a strong possibility, given the preferential association of different genomic regions with similar chromatin signatures.

It is also conceivable that the spatial organization of the nucleus, as well as the local chromatin landscape, impacts the various genome activities that rely on DNA transactions, such as transcription, replication, DNA repair, and recombination [72]. Thus, the epigenome and the transcriptome may affect genome replication dynamics. One of the primary regulatory steps of genome replication is the specification of ORIs. As discussed above, it will be important to identify the originome, which is the collection of all ORIs active in a plant. Efforts to achieve this still face difficulties derived from the limited amounts of short nascent DNA strands purified from replication bubbles and the complexity of the analysis. A future step should aim to identify possible differences in the originomes of specific cell types. This will be a major advance in this field that will open various experimental possibilities to establish links between the originome, the epigenome, and the transcriptome. Systematic and comprehensive studies on these aspects, and surely others, should reveal the mechanisms that relate chromatin and nuclear organization with developmental processes, hormonal responses, and environmental challenges. We look forward to these and many other exciting achievements in this field.

## Box 1. Names of the genes mentioned in this review

*ABO4* = *ABA OVERLY SENSITIVE 4*

*AG* = *AGAMOUS*

*AGO4* = *ARGONAUTE 4*

*ATR* = *ATAXIA TELANGIECTASIA-MUTATED AND RAD3-RELATED*

*ATXR5* = *ARABIDOPSIS TRITHORAX-RELATED PROTEIN 5*

*ATXR6* = *ARABIDOPSIS TRITHORAX-RELATED PROTEIN 6*

*CAF-1* = *CHROMATIN ASSEMBLY FACTOR-1*

*CLF* = *CURLY LEAF*

*CMT2* = *CHROMOMETHYLASE 2*

*CMT3* = *CHROMOMETHYLASE 3*

*CP190* = *Centrosome-associated zinc finger protein 190*

*CTCF* = *CCCTC-binding factor*

*DDB2* = *DNA DAMAGED BINDING PROTEIN 2*

*DDM1* = *DECREASED DNA METHYLATION 1*

*DPB2* = *DNA POLYMERASE EPSILON SUBUNIT B2*

*DRM2* = *DOMAINS REARRANGED METHYLTRANSFERASE 2*

*EIN3* = *ETHYLENE INSENSITIVE 3*

*EMF2* = *EMBRYONIC FLOWER 2*

*ESD7* = *EARLY IN SHORT DAYS 7*

*FEN1* = *FLAP ENDONUCLEASE I*

*FLC* = *FLOWERING LOCUS C*

*FRIGIDA* = *FLOWERING LOCUS A*

*FT* = *FLOWERING LOCUS T*

*HP1* = *Heterochromatin Protein 1*

*IBM1* = *INCREASE IN BONSAI METHYLATION 1*

*ICU2* = *INCURVATA 2*

*INO80* = *INOSITOL AUXOTROPHY 80*

*KYP* = *KRYPTONITE*

*LDL2* = *LYSINE-SPECIFIC DEMETHYLASE LIKE 2*

*LHP1* = *LIKE HETEROCHROMATIN PROTEIN 1*

*MET1* = *METHYLTRANSFERASE 1*

*MOP1* = *MEDIATOR OF PARAMUTATION 1*

*MOP3* = *MEDIATOR OF PARAMUTATION 3*

*MSI* = *MULTICOPY SUPRESSOR OF IRA*

*NAP1* = *NUCLEOSOME ASSEMBLE PROTEIN 1*

*NRP* = *NAP1-RELATED PROTEINS*

*NRP1* = *NAP1-RELATED PROTEIN 1*

*POLA1* = *DNA POLYMERASE ALPHA 1 CATALYTIC SUBUNIT*

*POLD1* = *DNA POLYMERASE DELTA 1 CATALYTIC SUBUNIT*

*POLD2* = *DNA POLYMERASE DELTA 2 ACCESSORY SUBUNIT*

*POLE1* = *DNA POLYMERASE EPSILON 1 CATALYTIC SUBUNIT*

*PRC2* = *POLYCOMB REPRESSIVE COMPLEX 2*

*RAD51* = *RADIATION SENSITIVE 51*

*RDR2* = *RNA-DEPENDENT RNA POLYMERASE 2*

*ROS1* = *REPRESSOR OF SILENCING 1*

*SEP3* = *SEPALLATA 3*

*SET/TAF-1β* = *SET/template-activating factor-1β*

*SOC1* = *SUPPRESSOR OF OVEREXPRESSION OF CONSTANS 1*

*SOG1* = *SUPPRESSOR OF GAMMA RADIATION 1*

*Swi6* = *Switching deficient 6*

*SWR1* = *Swi2/Snf2-related 1*

*TIL1* = *TILTED 1*

*TREX* = *Transcription-coupled Export*

*WEE1* = *WEE1 KINASE HOMOLOG*

## Abbreviations

3D: Three-dimensional
DDR: DNA damage response
DSB: Double-strand DNA break
HR: Homologous recombination
ORI: DNA replication origin
RdDM: RNA-dependent DNA methylation
siRNA: Short interfering RNA
TAD: Topologically associating domain
TE: Transposable element
TSS: Transcription start site
